# Examination of a Mechanical Amplifier in the Incudostapedial Joint Gap: FEM Simulation and Physical Model

**DOI:** 10.3390/s140814356

**Published:** 2014-08-07

**Authors:** Martin Koch, Till Moritz Eβinger, Matthias Bornitz, Thomas Zahnert

**Affiliations:** Clinic of Otorhinolaryngology, Department of Medicine, Technische Universitat Dresden, Universitätsklinik Carl Gustav Carus, Fetscherstraβe 74, 01307 Dresden, Germany; E-Mails: tillmoritz.essinger@uniklinikum-dresden.de (T.M.E.); Matthias.Bornitz@uniklinikum-dresden.de (M.B.); Thomas.Zahnert@uniklinikum-dresden.de (T.Z.)

**Keywords:** fully implantable, membrane transducer, ossicular chain, middle ear, hearing aid, FEM, feedback suppression

## Abstract

Implantable assembly components that are biocompatible and highly miniaturized are an important objective for hearing aid development. We introduce a mechanical transducer, which could be suitable as part of a prospective fully-implantable hearing aid. The transducer comprises a sensor and an actuator unit in one housing, located in the joint gap between the middle ear ossicles, the incus and stapes. The setup offers the advantage of a minimally invasive and reversible surgical solution. However, feedback between actuator and sensor due to mechanical coupling limits the available stable gain. We show that the system can be stabilized by digital control algorithms. The transducer is tested both in a finite elements method simulation of the middle ear and a physical model of a human middle ear. First, we characterize the sensor and actuator elements separately. Then, the maximum stable gain (MSG) of the whole transducer is experimentally determined in the middle ear model. With digital feedback control (using a least mean squares algorithm) in place, the total signal gain is greater than 30 dB for frequencies of 1 kHz and above. This shows the potential of the transducer as a high frequency hearing aid.

## 1. Introduction

### 1.1. Implantable Hearing Aids

The role of implantable hearing aids as a treatment for hearing loss is increasing. Besides visual advantages, they provide the benefit of a free ear canal and a more natural signal acquisition. Several active implants for the treatment of intermediate hearing loss have been developed to serve as an alternative for conventional hearing aids. The references [1–3] give an overview of existing middle ear implants. It should also be noted that for severe inner ear pathology, the current state-of-the-art treatment is the cochlear implant, which applies an electrical stimulus directly on the auditory nerve. However, in this study, we will focus on the acoustic or mechanical amplification type of implantable hearing aid. Devices of this sort usually consist of a microphone (sensor), a receiver (speaker or actuator) and a digital signal processor. They are either partially or fully implantable. The former category usually implies that the actuator is implantable and the sensor (microphone) is placed outside the ear. Examples of this type of implant are the widely used Vibrant Soundbridge (MedEl Austria) or the Soundtec Direct Drive (Soundtec Inc.). In both of these, the actuator is not fixed to the tympanic cavity, but is clamped onto the ossicular chain as a free-floating mass. If both the microphone and receiver are implanted, the system is called fully implantable. Examples include the Carina (Otologics) and the Esteem (Envoy Medical Corp.). The actuator in these systems is usually permanently fixed in the tympanic cavity. This provides the device with mechanical support, but requires complex surgery during implantation. All currently known implants are designed with separate housings for the microphone and receiver. This is done so as to acoustically and mechanically insulate the sensor from the actuator and to minimize feedback.

### 1.2. Implantable Transducer inside the Incudostapedial Joint Gap

**Figure 1.**
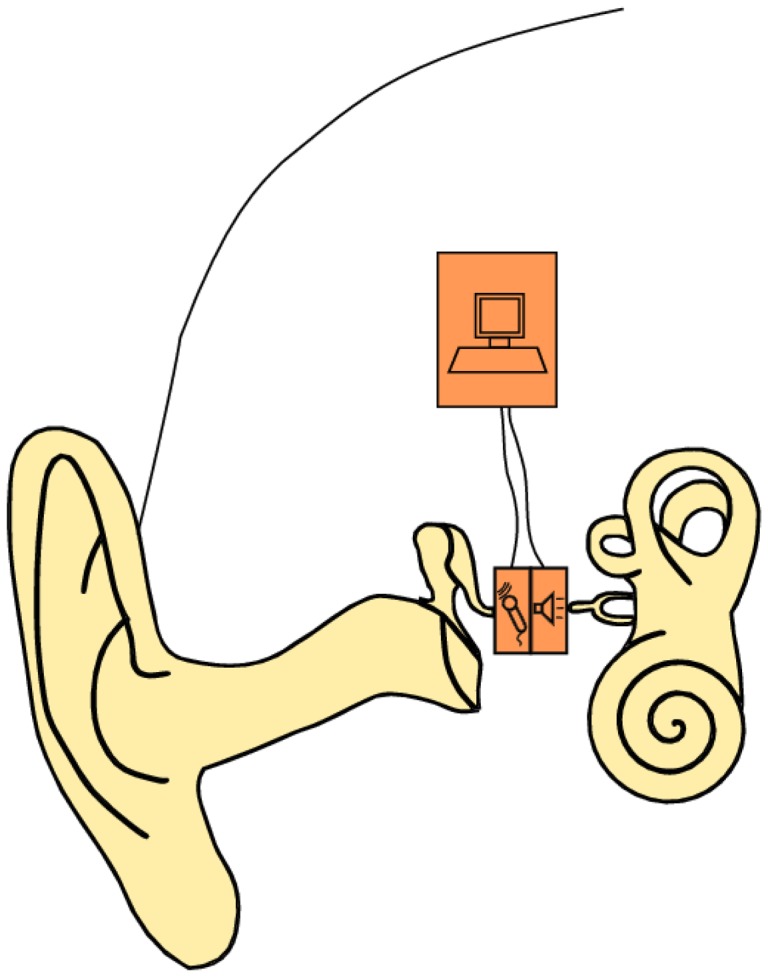
Transducer schematic diagram.

**Figure 2.**
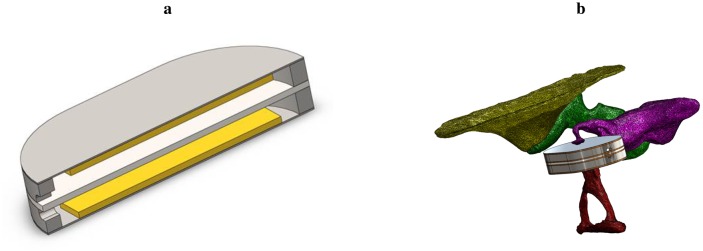
Transducer in the incudostapedial joint gap. (**a**) Cross-sectional view of the examined transducer; (**b**) transducer position in the tympanic cavity.

In this study, we present a bidirectional membrane transducer to be used as an acousto-mechanical amplifier in the middle ear, as shown schematically in Figure 1. A cross-sectional view of the transducer is shown in Figure 2a. It is assembled from two identical oval-shaped titan housings mounted back to back, creating a single frame with two separate air-filled cavities. They are capped with a very thin membrane each. A piezoelectric element is glued to the inside of each membrane. They act as bending plates, one for signal acquisition (sensor) and one for signal generation (actuator). The oval-shaped transducer measures 4 mm × 2.5 mm with a thickness of 1 mm. It is inserted into the ossicular chain at the joint gap between the long process of the incus and the stapes head (see Figure 2b). Following a mastoidectomy (the surgical access to the middle ear used for most such implants), the joint is opened by the surgeon. This is a minimally invasive surgery that has been shown to be reversible [4]. The transducer is then slid into the joint and is held in place by adhesive forces and the tension in the ossicular chain. The piezo sensor thus detects the vibrations of the tympanic membrane through the ossicles and converts these into an analogous voltage signal, which is processed and amplified. The amplified voltage signal drives the piezo actuator and moves the stapes, providing enhanced acoustic stimulus to the inner ear. For the purpose of this study, the necessary signal processing and control software is conducted on a computer with a field programmable gate array (FPGA) card. Prospective prototypes will likely feature implanted or external signal processors similar to those used in cochlear implants.

### 1.3. Aims of the Study

The proposed transducer design offers distinct advantages in implantation simplicity and time. However, due to the direct mechanical coupling between sensor and actuator, strong feedback can be expected. Furthermore, the actuator is not braced to the larger structure of the tympanic cavity. This raises the question of how well the actuator is able to transfer its movement into the stapes, knowing the relatively high acoustic impedance of the liquid-filled inner ear. In this paper, we will examine the proposed device and the surrounding structures both in finite elements method (FEM) simulation and in a physical model of the human ear. The results will be compared and collated with existing literature. We aim to show how well the device can be stabilized with digital feedback suppression, determine its dynamic working range as a hearing aid and predict a possible medical indication for its application.

## 2. Method and Materials

### 2.1. Simulation

**Figure 3.**
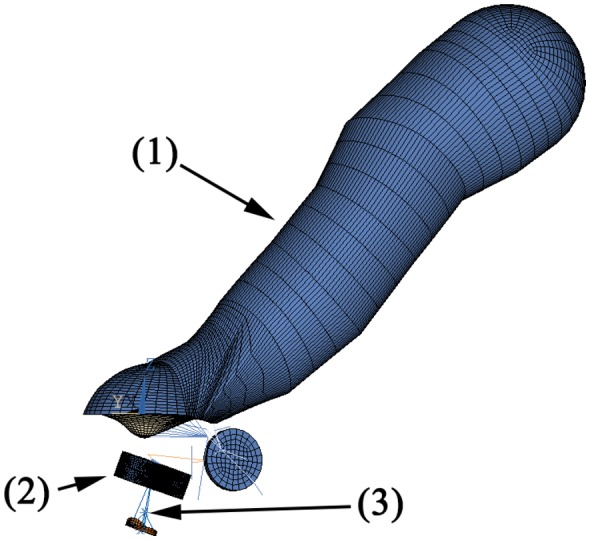
FEM model of the middle ear with the ear canal (1) and transducer (2) inside the ossicular chain just above the stapes (3).

The FEM simulation model of the human middle ear used in this study has been described in [5]. The model is illustrated in Figure 3 and consists of:

- the ear canal as acoustic fluid with a third order boundary condition at the entrance for the simulation of the surrounding air
- the eardrum as orthotrop-elastic shell elements with a constant damping ratio
- the ossicles as rigid bodies
- the ligaments as elastic bars
- the joints as elastic bodies with a constant damping ratio
- the cochlea as a simplified spring-mass-damper model

The transducer housing is made of 3D-20-node solid elements. The sensor/actuator membranes and piezo crystal are built with 3D-20-node-coupled field elements with electromechanical coupling. According to [6], the mean stapes head diameter is 1.14 mm by 0.83 mm. In this simulation, we assume a circular contact area with a diameter of 1 mm at the stapes head, which is coupled with MPC-rigid184 elements to the attached structure. The simulation is calculated by harmonic analysis with 10 frequency points for up to 5 kHz. The excitation is either a pressure of 1 Pa at the eardrum (equal to 94 dB SPL or sound pressure level) or a voltage of 1V at the actuator. The resulting data is either the sensor voltage or the movement of the stapes footplate at the end of the ossicular chain. Note that with a linear model, only the open loop can be simulated. The closed loop system (which includes feedback from the actuator to the sensor) is therefore not examined in this simulation. However, due to the mechanical coupling between sensor and actuator, the closed loop system should be expected to exhibit strong feedback. The simulation data will therefore yield an approximate operational range. The measured data is expected to locally deviate from this due to closed loop resonances. The simulation model is used to determine:

- the middle ear transfer function of the ossicular chain with the implanted transducer compared to the intact ossicular chain
- the sensitivity of the sensor to air pressure at the ear drum (in mV Pa^−1^)
- the output of the actuator in the movement of the stapes footplate (in mm Pa^−1^), used as a measure for hearing impression (in dB SPL equivalent).

The model is also used to compare the idealized impedances of the ossicular chain at the incudostapedial joint before and after insertion. The former contact partners at the joint, long incus process and stapes head are both excited with a lateral force of 1N, and the resulting frequency-dependent ossicular movement is compared. Subsequently, the transducer is inserted, and the actuator is excited with a voltage of 1 V. The ossicular movement on both sides of the transducer (long process of the incus and stapes head) is recorded and compared. This serves to evaluate the effectiveness of the transducer concept and to quantify the drawbacks of the free-floating design.

### 2.2. Physical Model of the Middle Ear

**Figure 4.**
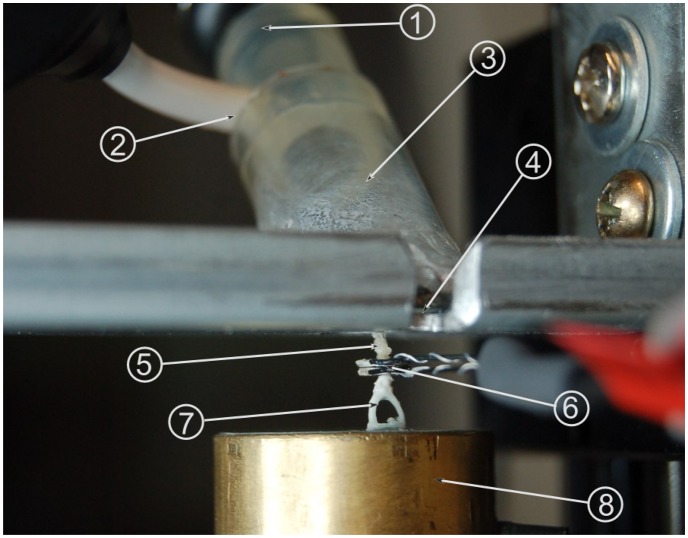
Setup of the experiment: (1) loudspeaker as the excitation source at the entrance to the model ear canal; (2) reference microphone; (3) acrylic glass model of ear canal; (4) silicone model of the tympanic membrane; (5) partial ossicular replacement prosthesis (PORP) and long process of human incus; (6) bidirectional transducer; (7) resin cast stapes replica; (8) inner ear impedance model.

In order to perform measurements on the transducer, a partial physical model of the human ear was devised. This provides several advantages compared to measurements in a temporal bone: The model is stable over time, which simplifies measurements. Furthermore, the physical model offers reproducible results, while the transfer characteristics of different temporal bones vary at ±10 dB [7]. This variation and other factors, such as anatomy contact points, can be neglected for the principal considerations in this study. The full range of characteristics can be examined in prospective temporal bones studies. The model was adapted from a tympanoplasty training model [8]. It is shown in Figure 4 and features an ear canal 1 cm in diameter made of acrylic glass, which sits at an angle of 45° relative to the model's tympanic membrane. The membrane is made from silicone foil, which has previously been determined to exhibit sound transfer characteristics similar to a real human tympanic membrane. The silicone membrane is fixed between a steel plate and a magnetic foil of 0.8 cm thickness, both of which are cut out at an oval shape, creating a tympanic membrane model of 12 mm length and 1 mm width. The aforementioned ear canal is glued to the magnetic foil. It also features a microphone in order to record the pressure at the tympanic membrane, which serves as the reference (input) signal. A headphone loudspeaker at the entrance to the ear canal is used as the excitation source. Attached to the center of the silicone membrane is a PORP (partial ossicular replacement prosthesis). Contrary to its normal usage, the PORP serves as a replacement for the malleus, which was hard to build in the model environment. The clamp at the stapes side of the PORP has been replaced with the long process of an incus. The steel plate is attached to a linear three-axle positioning slide. This allows for the positioning of this upper part of the setup at a resolution of 1 μm. The lower part of the setup begins with a vacuum-cast resin replica of a real stapes. The stapes footplate is attached to a Kapton film membrane simulating the annular ligament. The membrane is set right above one of two separate cavities within a hollow brass cylinder. There is another similar membrane at the top of the second cavity. Two microphones record the pressure inside the cavities separately. This is done so as to separate the air sound transfer from the sound transfer through the ossicular chain: by subtracting the microphone signals, the sound transferred through the air is negated in the measurement, as this is present in both cavities. This leaves only the sound transferred through the ossicles as the measured (output) signal. The subtraction of signals is performed by a differential amplifier.

### 2.3. Experimental Setup and Procedure

The device to be examined here consists of two identical piezoceramic single crystal elements connected by a solid titanium metal frame. It is inserted into the model of the middle ear described above. One of the piezoceramic elements serves as a mechanical sensor. As shown in Figure 2, this side touches the model of the incus and transduces the mechanical vibrations into an analogue voltage signal. The other piezoceramic element serves as a mechanical actuator that drives the movement of the stapes. Note that the device is not firmly attached to the surroundings, but is a free-floating addition to the ossicular chain. The inertial mass of the device is about 35 mg. The sensor and actuator are connected to the analogue input terminals of an FPGA (NI PXI-7842, National Instruments) via fourth order Butterworth filters (VPF 8 mk4, KEMO) for anti-aliasing and impedance matching. The analogue inputs of the FPGA have a dynamic range of ±10 V; the corresponding AD-converters feature 16-bit resolution. This calculates to a voltage resolution of:

(1) $$\Delta u=\frac{20\text{V}}{2^{16}}\approx 0.000305\text{V}$$

As will be shown later (see Section 3), the sensor's thermal noise is much lower than that. In order to make use of the sensor's dynamic range, this signal is therefore preamplified by a factor of 1000 (SR560 Low-Noise Preamplifier, Stanford Research Systems) prior to AD-conversion. Real-time signal processing and control algorithms are written in LabView (version 2011, National Instruments) and implemented on the FPGA. The internal resolution for the control algorithms is 16 bit fixed-point. It includes a variable low-pass filter (fourth order Butterworth filter), which is implemented through the use of the “Butterworth Filter express VI”, which is part of the LabView signal processing package. The central control algorithm is a 100-tap LMS adaptive filter as described above. A similar setup of hardware and algorithms has previously been successfully tested on a 10:1 scale model of the proposed device [9]. A loudspeaker at the entrance to the model ear canal serves as the stimulation source. The setup furthermore features a microphone each in the ear canal and in the inner ear behind the stapes footplate. Stimulation signals were generated and measurements were performed with a data acquisition board (NI PXI-4496, National Instruments). The software for measurement and signal generation was programmed in LabView (version 2011, National Instruments). The stimulation signal was either a 2048-point broadband multisine or a discrete frequency single sine signal. The physical model used here has shown itself to be valid for up to about 2.5 kHz. The sampling rate for both measurement and control software was 10 kHz, which is sufficient in this case. As stated in [9], it is sensible to use greater oversampling for the range beyond 2.5 kHz. However, a higher sampling rate would only shift the resonance frequencies further toward the outside of the desired audiological frequency range. This would actually have a positive impact on system stability, since these resonances could be attenuated without compromising hearing gain. The results obtained for this higher range are therefore expected to be valid as far as feedback control is concerned. For each measurement, five signals were recorded:

(1) Stimulus
(2) Ear drum pressure
(3) Sensor signal
(4) Actuator signal (generated)
(5) Inner ear pressure

**Figure 5.**
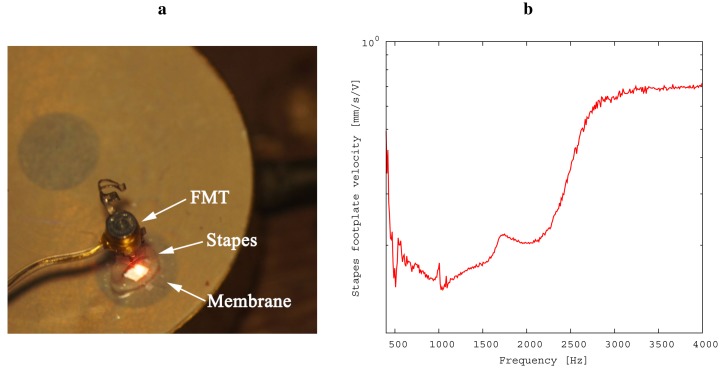
Calibration of the inner ear microphone. A floating mass transducer (FMT) excites the stapes **(left).** The velocity of the footplate is recorded by laser vibrometry along with the pressure inside the inner ear model cavity below the membrane. (**a**) Setup for calibration; (**b**) resulting calibration data.

Prior to conducting the experiment, the relationship of inner ear pressure to the velocity of the stapes footplate was determined (see Figure 5) in the following way: First, the model was separated at the incudostapedial joint; second, a floating mass transducer (FMT, as used in the Vibrant Soundbridge implant) was fixed at the stapes head for excitation; Third, simultaneous recordings of stapes footplate movement and inner ear pressure were conducted by laser Doppler vibrometry and by microphone, respectively. From this data, we were able to obtain a calibration data set for the microphone. This was necessary for practical reasons: The movement of the stapes footplate was not accessible directly during the experiment because of the setup geometry. Please note that the values for stapes footplate velocity shown in Section 3 were thus calculated from the measurements of the inner ear pressure. The following steps were taken in measuring:

**Intact ossicular chain:** The frequency response of the intact ossicular chain model (without the transducer in place) was recorded by stimulation of the loudspeaker. This serves as a reference for the analysis of the device's performance. Since the transducer is not inserted at this point, the only signals of interest are the two microphone signals at the ear drum and in the inner ear.
**Insertion of transducer:** The ossicular chain model was separated at the stapes head, and the transducer was inserted there. Again stimulating the loudspeaker, the frequency responses were recorded with the transducer in idle mode. The main points of interest in this measurement were the frequency-dependent sensitivity of the sensor to pressure at the eardrum and the attenuation of sound transfer along the ossicular chain due to the insertion of the transducer in V Pa^−1^.
**Noise:** Disconnecting the excitation source, the noise level of the device components was measured. From this measurement, the sensor's hearing threshold can be characterized.
**Actuator performance:** The actuator is stimulated with a 1V excitation. Measurements include the feedback path to the sensor, which we will compare to the simulation and to the frequency response of the converged adaptive filter described in Section 2.4. The main point of interest, however, is the induced movement of the stapes, which shows the upper bounds for the transducer's amplification. Note that as a free-floating device, the transducer has only its inertial mass to support it. The transfer function of actuator voltage to induced stapes velocity is therefore expected to drop at lower frequencies.
**Functional gain:** The loudspeaker is stimulated so as to produce a sound pressure level of 50 dB SPL at the ear drum, while measuring the velocity of the stapes footplate. This is done first with the transducer idle, then with the transducer set to a gain that offers a maximum signal yield for the stapes footplate movement without visible distortions and without resonance peaks caused by feedback.

### 2.4. Feedback Control

The theoretical amplification limit for the transducer is given by the sensitivity of the sensor and the footplate amplitude (or velocity) when the actuator is maximally excited. However, as has been noted above, the device is free floating. With only the inertial mass of the frame to support it, the momentum introduced by the actuator is divided between the movement of the stapes footplate and the recoil of the frame. Since the sensor is likewise attached to the frame, this causes a closed feedback loop in the system. It is well known that in electroacoustic feedback loops, the maximum available gain is limited, because the closed-loop system will begin to oscillate when the maximum stable gain (MSG) is exceeded [10]. Let *μ*(*ω*) be the forward path transfer function. If we define:

(2) $$G=\frac{1}{2\pi }\int_{0}^{2\pi }\left|\mu \left(\omega \right)\right|\text{d}\omega$$

as the broadband gain factor; the MSG is defined as the highest scalar value of *G* for which the system is stable. Suppression of feedback resonances smoothens the loop gain in the frequency domain and, thus, allows for a higher MSG value. In feedback applications, it is customary to refer to the MSG increase as a measure of the efficiency of the system. The Nyquist stability criterion states that the resonances or critical frequencies are located specifically at those frequencies for which the phase shift is a multiple of *2*π radians, which is a function of the time delay Δ*t_FB_* along the feedback path. MSG is reached when the gain for a critical frequency is increased to unity or beyond. Let us assume the mechanical coupling through the frame to be the dominant feedback path. The small measurements of the device and the solid metal frame lead to very short signal paths and, hence, high frequency resonances. The digital part of the forward path requires a sampling of the sensor signal at a finite sample rate, introducing a time delay *T* = 1/*fs*. Such a system cannot oscillate above the Nyquist frequency *fs*/2. Therefore, if T ≫ Δ*t_FB_*, the original resonances vanish, and new critical frequencies are introduced in the high end of the desired frequency range [9]. The system features an adaptive algorithm for digital feedback control based on the well-known least mean squares (LMS) algorithm first introduced by Widrow [11]: An adaptive filter is used to approximate the impulse response (IR) of the feedback path. Discrete convolution of the output (actuator) signal with the approximated IR yields an approximation of the feedback amplitude for a given input sample. This value is subtracted from the input signal before further processing is conducted. The adaptive algorithm requires an error signal, which is to be minimized. The sensor signal itself can be used as the error signal, provided that the input signal is transient. Let us assume that the forward path transfer function is perfectly flat in the desired frequency range. Let *F*(*ω*) be the feedback path transfer function and *F̂*(*ω*) its approximation. The transfer function for the closed loop:

(3) $$\frac{Y\left(\omega \right)}{X\left(\omega \right)}=\frac{G}{1-G\left(F\left(\omega \right)-\hat{F}\left(\omega \right)\right)}$$

approaches a constant value for a good approximation *F̂*(*ω*) of *F*(*ω*):

(4) $$\frac{Y\left(\omega \right)}{X\left(\omega \right)}\overset{\hat{F}\left(\omega \right)\to F\left(\omega \right)}{\to }G$$

In the ideal case of a perfect approximation *F̂*(*ω*) = *F*(*ω*), feedback is completely suppressed and *G* can take any value. The feedback control algorithm used here uses a 100-tap FIR (finite impulse response) filter for adaptive approximation. This corresponds to a total length of 10 *ms* at a sample rate of 10 *kHz*.

## 3. Results

**Figure 6.**
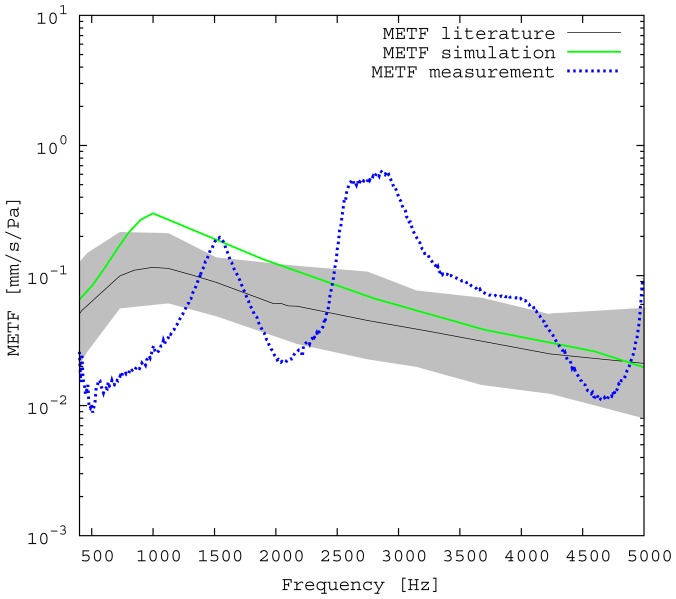
Comparison of simulated and measured middle ear transfer functions (METF) with literature data reproduced from [12]. Note that the measurements for the physical model are valid for frequencies below 2.500 Hz.

**Figure 7.**
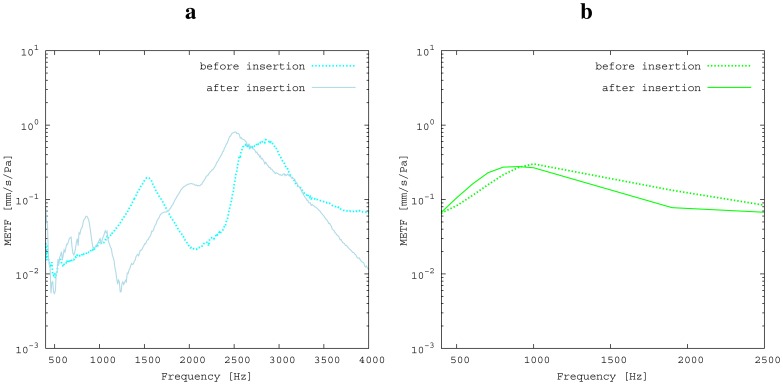
Influence of transducer implantation on middle ear transfer function (METF). The insertion of the additional mass into the ossicular chain primarily causes a shift in resonance peaks. (**a**) Measurement; (**b**) simulation.

**Figure 8.**
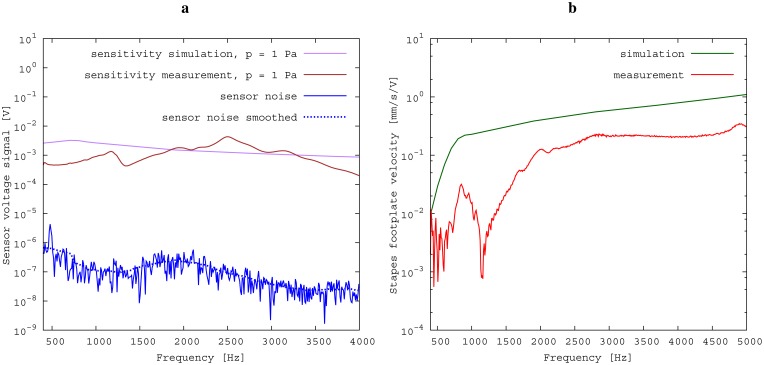
Performance of transducer assembly components. With the transducer in idle mode, the sensor signal for an excitation of 1 Pa at the eardrum is simulated, measured and compared to the sensor's thermal noise. A voltage of 1V is applied to the actuator, and the resulting velocity of the stapes footplate is determined. (**a**) Sensitivity of the sensor; (**b**) excitation of the actuator.

**Figure 9.**
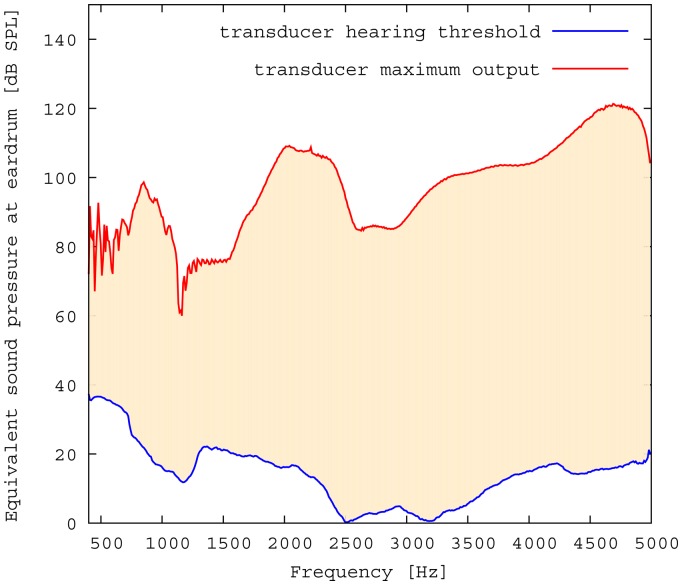
Dynamic range of the transducer in the model.

**Figure 10.**
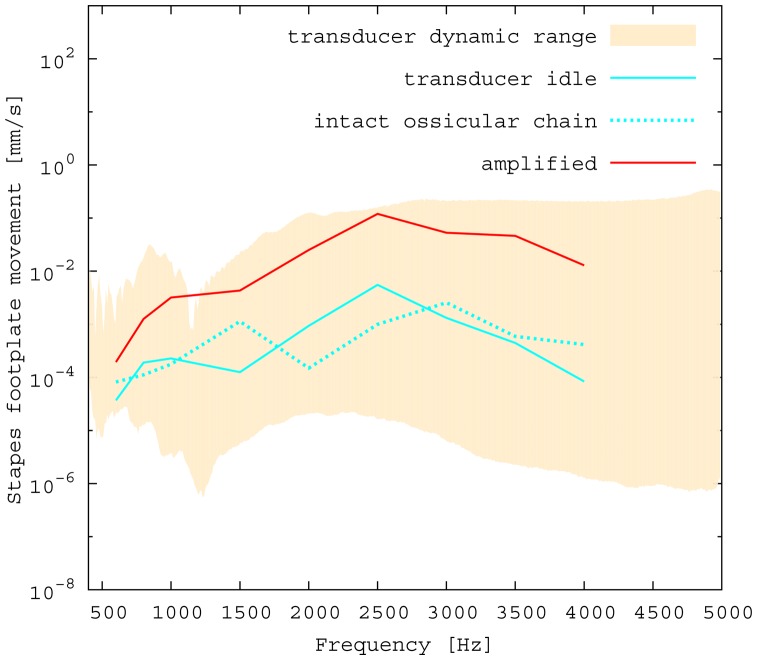
Performance of the transducer as an acoustic amplifier in the model. In addition to the original and the amplified signal, the frequency response for the intact ossicular chain is shown (dotted line) for comparison.

**Figure 11.**
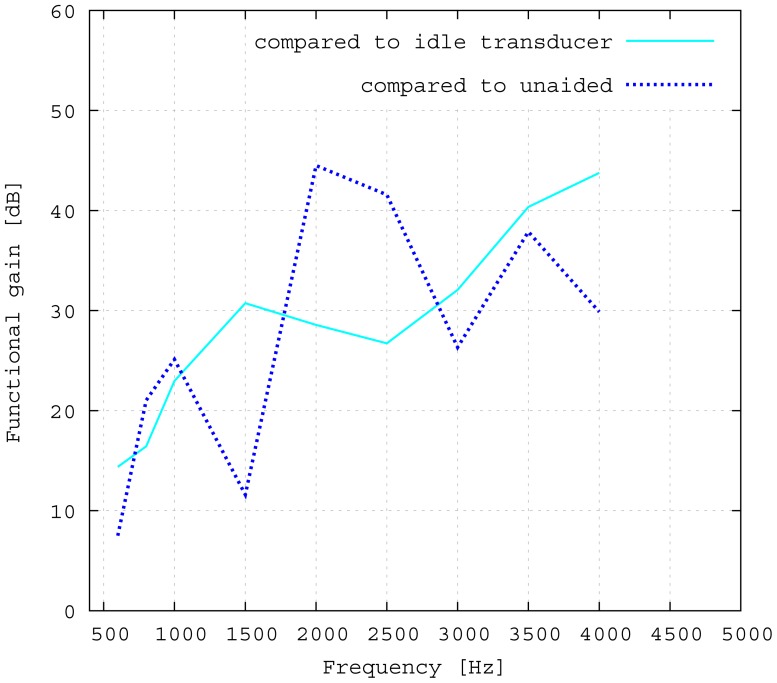
Achieved functional gain of stapes footplate velocity, as compared to both the implanted transducer in idle mode and the unaided case (with an intact ossicular chain).

The measured middle ear transfer function (METF) of the physical model and the simulated METF, as well as the literature data [12] are shown in Figure 6. Inserting the (idle) transducer into the ossicular chain affects the METF, as shown in Figure 7. The performance of the individual parts of the transducer is visualized separately for both the sensor and actuator element in Figure 8. The sensor in this setup has a sensitivity of around 1 mV for an excitation of 1 Pa pressure at the eardrum. The measured sensor noise and the measurement of the stapes footplate movement during the sensitivity-measurement are used to calculate the sensor's hearing threshold. If the actuator is excited with *U* = 1V, the measured movement of the stapes footplate is up to 3 × 10^−1^ mm s^−1^ V^−1^, which conforms to an equivalent sound pressure level of 120 dB SPL for this model. The dynamic range of the transducer can be derived by combining the characteristics of both the sensor and actuator. Figure 9 shows the dynamic boundaries of the system in equivalent decibel sound pressure level (dB SPL) at the ear drum. Theoretically, the transducer is able to amplify input signals at the eardrum of 25 dB SPL and below to a stapes movement, which equals the input pressures of 60 dB SPL up to 120 dB SPL. However, this is limited by the maximum stable gain available due to feedback between the actuator and sensor. For the given model using the previously described feedback suppression algorithms, a gain corresponding to a 29.5 dB amplification of the sensor signal could be adjusted. The system exhibits linear behavior within the physical limitations of the components. The amplified stapes footplate movement for a 50 dB SPL input signal is shown in Figure 10. This is compared to the intact ossicular chain (unaided case) and to the footplate movement with the idle transducer, as shown in Figure 11. For low frequencies up to one kilohertz, the transducer achieves amplifications of stapes footplate movement of 10 to 25 dB; in the high frequency range the sensor achieves a functional gain of 30 dB and more.

## 4. Discussion

### 4.1. Validation of Simulation and Model

The limitations of the FEM-simulation model used here have already been discussed in detail in [5]. As a synopsis, it can be said that the fact that the model is valid for linear computations only limits the maximum amplitude to 120 dB SPL. Regarding [13], the use of rigid bodies for the ossicles limits the validity of the model's frequency range to up to 3.5kHz. According to [14], the lack of air volumes in the model causes an inaccuracy of 5 dB, which can be neglected for fundamental studies, because it is below the natural distribution of middle ear transfer characteristics [7]. In the frequency range of 2.5 kHz and below, the measured frequency response for the physical model (Figure 6) matches the literature data well and seems to give a rough approximation for a typical middle ear behavior. The strong resonance between 2.5 and 3 kHz is uncharacteristic for normal METFs. Note that the literature range is an average of different METFs. It therefore does not exhibit distinct resonance peaks, such as those seen in the METF simulated for one specific middle ear. Inserting the (idle) transducer into the ossicular chain affects the METF, as shown in Figure 7. The simulation shows a down-shift of the resonance frequencies. This is to be expected due to the inertial mass added to the ossicular chain. The measurement shows a similar behavior, but also experiences additional distortions. It can be concluded that both the simulation model and the physical model can be used to obtain sufficiently accurate results for the frequency range below 2.5 kHz.

### 4.2. Evaluation of the Transferability to Temporal Bone Measurements

The results for the sensor element (Figure 8) can be confirmed by former investigations [15,16]. These previous studies were conducted on a sensor element of the same size as the whole transducer examined here. Results inside a calibration sound pressure box revealed a flat sensor frequency response. This suggests that the differences between measurement and simulation are mainly to be attributed to the different METFs. Previous studies have also revealed additional contact points in seven out of ten temporal bones. The impact on sensor output was found to be quite low (about 5 dB) [16]. The effect on the transducer sensor is expected to be on a similar scale. However, the effect of additional contact points on actuator output is unknown. Therefore, further studies for the transducer and especially its actuator element are necessary. The maximum stapes movement for the transducer in actuation mode with a voltage of *U* = 1 V is greater for the simulation than for the measurement at higher frequencies (see Figure 8). The difference is even greater at the anti-resonance of the model's METF with an inserted transducer at 1.2 kHz. (see Figure 7). The actuator is assumed to perform better in future setups in temporal bones, because the METF will resemble the simulation's METF more closely. The achievable functional gain of the model (Figure 10) seems to be only partly limited by system stability, but also by the maximum actuator output. This means that a higher actuator performance will conceivably result in a higher functional gain. The physical model and the simulation model are both focused on the linear piston-like mode of the stapes footplate movement. This is said to contribute to the the preeminent part for auditory sensation for lower frequencies up to 1 kHz ([17]) and still the dominant part for higher frequencies up to 4kHz ([14]). This comprises both the validated frequency range of the simulation model, as well as the physical model. However, because of the high impact of the actuator on the ossicular movement, the complex movement of the stapes can change in the clinical situation. If, e.g., the transducer is not positioned symmetrically, the influence of the rocking stapes motion on auditory sensation, which is described in [18], could be increased and would need attention. It must be noted that the setup examined here does not have a closed tympanic cavity volume. The following feedback paths are expected in prospective temporal bone experiments:

(1) mechanical feedback through the frame
(2) electromagnetic feedback
(3) acoustic feedback inside the transducer
(4) acoustic feedback inside the tympanic cavity
(5) body noise through the bone

In this study, the dominant path by far is the mechanical feedback through the frame (1). The first three paths are expected to be realistically represented by the physical model. Reflections and resonances in a closed volume should be expected to introduce additional feedback (4). To estimate the impact of these additional paths, both the magnitude and the phase of the feedback signal must be considered. As explained in Section 2.4, these correspond to the signal attenuation and delay along the paths, respectively. The length of these paths is about 2 cm in air, leading to a delay of ≈60 μs. This is still less than the sample interval *T* = 100 μs used here. The measurements presented here show that the compensation algorithms are well suited to handle such feedback delays. Furthermore, the sensor is designed as a force sensor for ossicular chain vibrations rather than an acoustic microphone. The coupling of sound from the cavity can therefore be expected to contribute only a minor part to the total feedback magnitude. The ossicular chain is connected to the temporal bone only through soft tissue, such as membranes, ligaments and fluids. The effect of feedback through body noise (5) can therefore be expected to be negligible. Further studies in the temporal bone should still be conducted.

### 4.3. Advantages and Drawbacks of the Transducer Design

**Figure 12.**
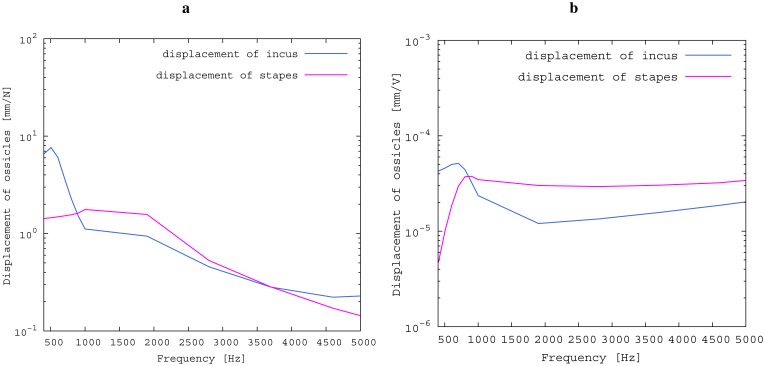
Excitation of the ossicles at the incudostapedial joint (FEM simulation). First, a force of 1N is applied to the opened incudo stapedial joint; second, the transducer is inserted, and a voltage of 1V is applied to the actuator. (**a**) Force on the opened joint; (**b**) excitation of the actuator.

The advantages of the transducer design without fixation in the tympanic cavity are primarily in terms of easy operability, surgery time and the reversibility of the surgery. All of these are very important characteristics of modern implants. From a technical point of view, rigid fixations in the tympanic cavity and a physical separation between the sensor and actor element appear to be preferable. In fact, all current hearing implants use either one or both of these approaches [1]. To assess the influence of the missing fixation, the impedances of the opened incudostapedial joint in the direction of both incus and stapes are simulated and illustrated in Figure 12. The movement response to a force (Figure 12a) is bigger in the incus direction than in the stapes direction for lower frequencies and similar for higher frequencies. If the transducer is inserted and the actuator is driven with *U* = 1 V, the incus movement is still much higher than the stapes movement for low frequencies up to 800 Hz (see Figure 12b). This matches the drop in the measured frequency response towards low frequencies (see Figure 11). In the ideal case of a complete fixation of the transducer to the temporal bone, there would be no movement of the transducer frame in the direction of the incus. Hence, the movement of the actuator plate would only drive the stapes side, with a frequency response for the desired stapes movement equal to the sum of the two curves in Figure 12b. This would clearly have a large impact on the low frequency output, but would add only about 2 dB above 1 kHz. Since the most common medical indication is high frequency hearing loss, the advantages of an easy, quick and reversible surgery seem to outweigh the disadvantages of the free-floating design. Combining the sensor and actuator in one assembly causes mechanical coupling, which results in strong feedback. However, as has been noted above, the signal paths are very short, causing high frequency feedback that lies, in fact, outside the desired frequency range. AD conversion in the forward path limits the signal to this frequency band and effectively cuts off the inherent critical frequencies, stabilizing the system. The remaining feedback can be controlled very well with the approach of adaptive primary path filtering. This has also been demonstrated in a larger scale physical model in [9]. An MSG increase of up to 29.5 dB is reported here, which compares very well with the current state-of-the-art in feedback suppression in hearing aids [19]. In the higher frequency range, added gain is not impeded as much by the aforementioned lack of inertial support. The functional gain achieved at frequencies greater than 1500 Hz is similar to the functional gain of existing hearing aids.

### 4.4. Medical Indication

**Figure 13.**
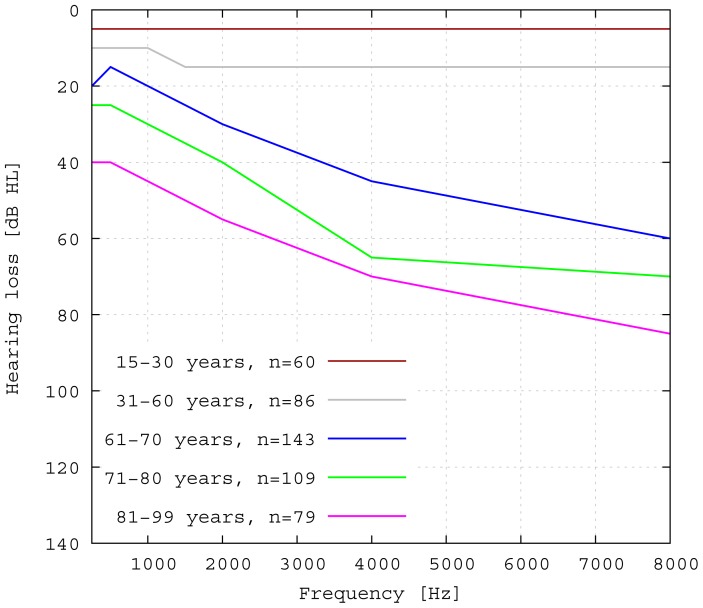
Statistical hearing loss for probands without known former ear diseases according to [20] in dB hearing level.

**Figure 14.**
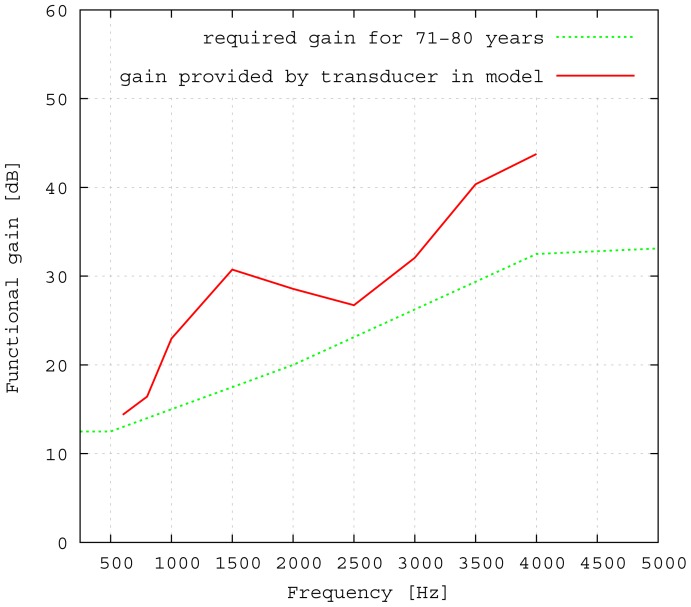
Required functional gain for hearing aids as calculated by the Berger method [22] and the functional gain provided by the proposed transducer.

The transducer is able to achieve a functional gain of 30 dB and more for higher frequencies. High frequency hearing loss is the most common kind of hearing impairment that occurs in elder people. Figure 13 shows the statistical hearing loss for people of different ages without known hearing diseases [20]. Note that the discomfort level does not rise in the same manner as the hearing loss ([21]). Therefore, the input signals for the transducer cannot be scaled linearly, but need to be compressed, which leads to a loss of dynamic range for hearing. Furthermore, the gain has to be fitted for each patient, due to the individual variations in hearing discomfort level. Estimations for a medical indication for the proposed transducer can be made from the commonly used gain fitting methods. For example, the Berger method proposes gains for different frequencies that are all near the half hearing loss [22]. In Figure 14, the required functional gain as calculated by the Berger method for a statistical mean of 71–80-year-olds is compared to the measured functional gain of the transducer. The comparison indicates that the transducer is appropriate for a large number of patients.

## 5. Conclusions

A fully-implantable hearing device in the form of a membrane transducer in the incudostapedial joint for hearing amplification is introduced. The transducer was evaluated in an FEM-simulation and in a physical model of the middle ear. The sensitivity of the transducers sensor element was around 1 mV per Pascal pressure at the eardrum. The sensor hearing threshold is 25 dB and below, the actuator maximum output equals up to 120 dB equivalent decibel sound pressure level at the eardrum. In the physical model, the achieved functional signal gain for the stapes footplate movement (which was used as a measure for auditory sensation) was greater than 30 dB for frequencies of 1 kHz and above. Comparisons with statistical hearing thresholds of elderly people without known hearing diseases show that the transducers amplification characteristics fit the most common hearing loss precipitated by aging quite well and seem to match the average hearing loss of 71–80-year-olds. This offers a big area of application for the presented transducer. Further research should be undertaken to confirm these results by measurements in human temporal bones. This could reveal additional unobserved influence factors of the more realistic environment. For example, the preload influence on the ossicular chain during transducer insertion or more complex three-dimensional stapes movements could require attention. Classification of the transducer performance by a stapes motion comparison as conducted in this study is the most important parameter for transducer evaluation. Nevertheless, more detailed parameters, like the absence of distortion, that are essential for speech understanding should be included in future transducer assessments.
